# A study of long-term GABA and high-energy phosphate alterations in the primary motor cortex using anodal tDCS and ^1^H/^31^P MR spectroscopy

**DOI:** 10.3389/fnhum.2024.1461417

**Published:** 2024-12-13

**Authors:** Harshal Jayeshkumar Patel, Lea-Sophie Stollberg, Chang-Hoon Choi, Michael A. Nitsche, N. Jon Shah, Ferdinand Binkofski

**Affiliations:** ^1^Division of Clinical Cognitive Sciences, Department of Neurology, RWTH Aachen University Hospital, Aachen, Germany; ^2^Institute of Neuroscience and Medicine-4, Forschungszentrum Jülich GmbH, Jülich, Germany; ^3^Leibniz Research Centre for Working Environment and Human Factors, Department of Psychology and Neurosciences, Dortmund, Germany; ^4^JARA-BRAIN-Translational Medicine, Jülich-Aachen-Research-Alliance (JARA), Aachen, Germany; ^5^Department of Neurology, RWTH Aachen University Hospital, Aachen, Germany; ^6^Institute of Neuroscience and Medicine-11, Forschungszentrum Juelich, Jülich, Germany

**Keywords:** tDCS, GABA, ^31^PMRS, ^1^HMRS, primary motor cortex, neuroplasticity, energy metabolism

## Abstract

**Introduction:**

Anodal transcranial direct current stimulation (tDCS) has been reported to modulate gamma-aminobutyric acid levels and cerebral energy consumption in the brain. This study aims to investigate long-term GABA and cerebral energy modulation following anodal tDCS over the primary motor cortex.

**Method:**

To assess GABA and energy level changes, proton and phosphorus magnetic resonance spectroscopy data were acquired before and after anodal or sham tDCS. In anodal stimulation, a 1 mA current was applied for 20 min, and the duration of ramping the current up/down at the start and end of the intervention was 10 s. In the sham-stimulation condition, the current was first ramped up over a period of 10 s, then immediately ramped down, and the condition was maintained for the next 20 min.

**Results:**

The GABA concentration increased significantly following anodal stimulation in the first and second post-stimulation measurements. Likewise, both ATP/Pi and PCr/Pi ratios increased after anodal stimulation in the first and second post-stimulation measurements.

**Conclusion:**

The approach employed in this study shows the feasibility of measuring long-term modulation of GABA and high-energy phosphates following anodal tDCS targeting the left M1, offering valuable insights into the mechanisms of neuroplasticity and energy metabolism, which may have implications for applications of this intervention in clinical populations.

## Introduction

Several studies have demonstrated molecular and neurophysiological evidence linking altered neuronal plasticity to neurological disorders (Di Lazzaro et al., 2010) and altered energy metabolism to metabolic disorders (Soares et al., 2011) in brain areas. It has been suggested that metabolic disorders are associated with neurological disorders (Crabtree and Gogos, 2014; Penninx and Lange, 2018). For instance, abnormal neuroplasticity mediated by altered neurotransmission (Harrison, 1999) has been observed in the prefrontal cortex in schizophrenia patients (Hulshoff Pol and Kahn, 2008). One suggested explanation is that hypofunction of the NMDA-type glutamate receptor cause a decrease in the excitation of GABAergic interneurons, resulting in glutamatergic neurons disinhibition (Kondziella et al., 2007; Moghaddam et al., 1997; Olney et al., 1999; Stone et al., 2009). This disinhibition of glutamatergic neurons may result in excessive glutamate stimulation, which may cause neuronal damage or death via excitotoxicity leading to a hyperdopaminergic state and psychosis. In an EEG study comparing healthy subjects with schizophrenia, variation in the P3a distribution was found which shows differences in the attention system activity (Mugruza-Vassallo and Potter, 2019). Functional imaging studies that assess regional cerebral blood flow (rCBF) can help identify cerebral activity associated with schizophrenia (Tamminga, 1999). For example, Liddle et al. (1992a) and Liddle et al. (1992b) found a negative correlation between the psychomotor poverty symptoms of schizophrenia and rCBF in the lateral prefrontal cortex and overactivity in the striatum.

Pharmacological studies have discovered genes involved in the pathophysiology of schizophrenia (De Jong et al., 2016; Gaspar and Breen, 2017; Rodriguez-Lopez et al., 2020; Ruby et al., 2014), such as GRM3 expression in astrocytic cells, which is required to protect neurons against NMDA induced neurotoxicity (Hikmah et al., 2023). This suggests that diminished GRM3 functionality, which may result in hyper-glutamatergic signalling, may cause more damage to the neurons as a result of the abnormal signaling exhibited in schizophrenia (Saini et al., 2017). In schizoaffective disorder, both schizophrenic and affective occur at the same time (Werner and Covenas, 2016). The mesolimbic system (the hippocampus and prefrontal cortex) plays an important role in the symptoms, which involves alteration in a multi-neurotransmitter system such as dopamine, serotonin, GABA and glutamate (Kulkarni et al., 2022) due to susceptibility genes such as neuregulin-1 dysbindin-1, GAD67 catechol-O-methyl transferase and monoamine oxidase (Haller et al., 2014; Ruby et al., 2014; Werner and Coveñas, 2013).

Several studies using magnetic resonance spectroscopy (MRS) have indicated that major depressive disorder (MDD) individuals have lower GABA concentrations in occipital cortex than healthy controls (Sanacora et al., 2004; Sanacora et al., 1999; Sanacora et al., 2003). MDD, along with GABA, is also related to altered serotonin activity. In many research studies, the binding potential of serotonin receptor, 5-hydroxytryptamine or (5-HT) was found to be lower in patients with MDD than in control subjects (Bhagwagar et al., 2004; Drevets et al., 1999; Hirvonen et al., 2008; Sargent et al., 2000). Selective serotonin reuptake inhibitors (SSRI) are most widely used to treat MDD and have shown significant change in the dorsolateral prefrontal cortex (DLPFC) volume and resting-state functionality (Lee et al., 2023).

Altered neurotransmission as seen in MDD is multifaceted and has been associated with the risk of developing severe medical disorders. i.e. it increases the risk of cardiovascular disorders by 1.5–2 fold (Van der Kooy et al., 2007) for stroke 1.8 fold (Ramasubbu and Patten, 2003) for Alzheimer’s disease by 2.1 fold (Green et al., 2003) and diabetes by 60% (Mezuk et al., 2008). The relationship between metabolic disorder and other psychiatric disorders is evident in the above-mentioned studies. Moreover psychiatric disorders are often accompanied by cognitive impairment in various domains, such as attention, executive functions, memory, and processing speed, as highlighted in studies (Grundman et al., 2004; McIntyre et al., 2015; Millan et al., 2012; Reichenberg, 2010). GABA neurotransmitter plays an important role in cognition and several studies have shown a link between dorsolateral prefrontal cortex GABA to working memory (Duncan et al., 2014; Yoon et al., 2016) and supplementary motor area GABA levels to motor distraction (Duncan et al., 2014; Mullins et al., 2014) and to alterations in cognitive processing with age (Harris et al., 2017; Porges et al., 2017). The interaction between altered neuroplasticity and metabolism has implications on cognitive functions or impairment. Therefore, simultaneously studying the neurochemical mechanisms behind neuronal plasticity and energy metabolism may offer significant breakthroughs for therapeutic interventions. With the advanced assessment of high-energy phosphate and neurotransmitters like gamma amino butyric acid (GABA) we can get a deeper insight into the mechanism of such changes in the brain of patients suffering from psychiatric and metabolic disorders.

A well-established approach for modulating neuronal plasticity and energy in humans is a non-invasive brain stimulation technique known as transcranial direct current stimulation (tDCS). By targeting specific brain regions, tDCS can be used to modulate brain energy metabolism and neuronal plasticity. Human *in vivo* studies have demonstrated that the application of anodal tDCS in the primary motor cortex can lead to spontaneous firing rates of cortical neurons, inducing neuronal excitation and altering energy consumption (Binkofski et al., 2011; Nitsche and Paulus, 2000). This modulation in excitability and energy is likely mediated by an altered concentration of the inhibitory neurotransmitter GABA (Stagg et al., 2009) and high-energy phosphates, e.g., adenosine triphosphate (ATP) and phosphocreatine (PCr).

Owing to ongoing advancements in magnetic resonance (MR) techniques (Choi et al., 2021; Henning, 2018; Wilson et al., 2019), changes in GABA and high-energy phosphates in M1 following tDCS can be monitored using MR spectroscopy (MRS). Specifically, it has been shown that proton (^1^H)-MRS (Patel et al., 2019) and phosphorus (^31^P)-MRS can be used to measure changes in GABA and energy phosphates in the M1, for example, following anodal tDCS as compared to sham tDCS. The time courses of separately measured concentrations of GABA and ATP/PCr in the aforementioned studies indicated that GABAergic activity and bioenergetics inside the M1 might work together to modulate neuronal excitability and energy. Consecutive measurement of both ^1^H- and ^31^P-MRS, before and after tDCS, could offer a promising approach to yield information related to plastic adaptation and energy consumption in both healthy and diseased brains.

In this study, we hypothesize that the application of anodal tDCS to the M1 region modulates GABA concentration and brain energy consumption. Notably, this work presents a measurement approach that integrates one pre-tDCS, ^1^H- and ^31^P-MRS measurement and two consecutive post-anodal tDCS, ^1^H- and ^31^P-MRS measurements to demonstrate the viability of using MRI and MRS to measure the long-term modulation of GABA and high-energy phosphates following anodal tDCS targeting the left M1 for the first time.

## Materials and methods

Forty-four healthy subjects (22 anodal and 22 sham) with no history of neurological or psychiatric diseases were recruited for this single-blind, randomized control pilot study. The subjects (Mean age: 28 ± 7; Gender: 26 Female and 18 Male) were all right-handed, as evaluated by the Edinburgh Handedness Inventory (Oldfield, 1971). All examinations were conducted at the Division of Clinical Cognitive Sciences of the RWTH Aachen University, Germany, and subjects provided their written informed consent for participation in this pilot study, which was agreed upon by the local ethics committee.

The non-invasive DC-Stimulator (neuroConn GmbH, Germany) used to stimulate the M1 region was programmed to deliver constant, direct current to the brain via two rubber electrodes (5 × 7 cm) covered by saline-soaked (0.9% NaCl) sponges, which modulate brain activity. One electrode was centered over the left M1 (5 cm lateral to Cz, C3) and the other over the contralateral supraorbital ridge using the conventional EEG 10/20 system. To accomplish the electrical contact between the electrodes and the scalp, 0.9% NaCl solution was used as a conducting medium. In anodal stimulation, a 1 mA current was applied for 20 min, and the duration of ramping the current up/down at the start and end of the intervention was 10 s. In the sham-stimulation condition, the current was first ramped up over a period of 10 s, then immediately ramped down, and the condition was maintained for the next 20 min.

**Figure 1:**
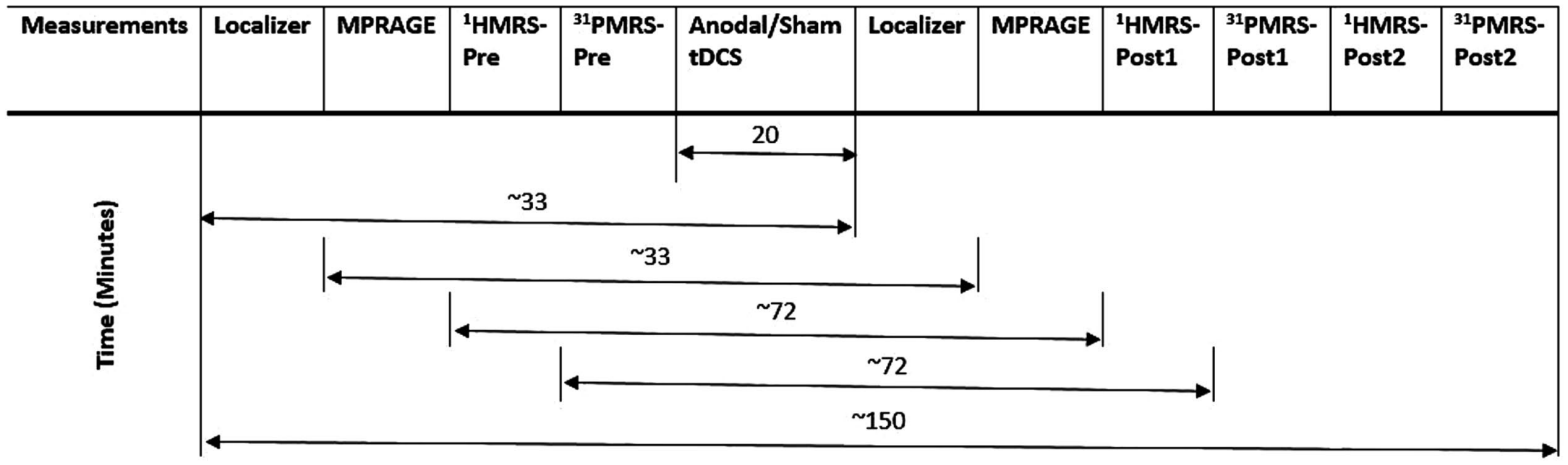
The table shows the ^1^H GABA, ^31^P MRS and other MR measurements performed before and after tDCS.

**Figure 2:**
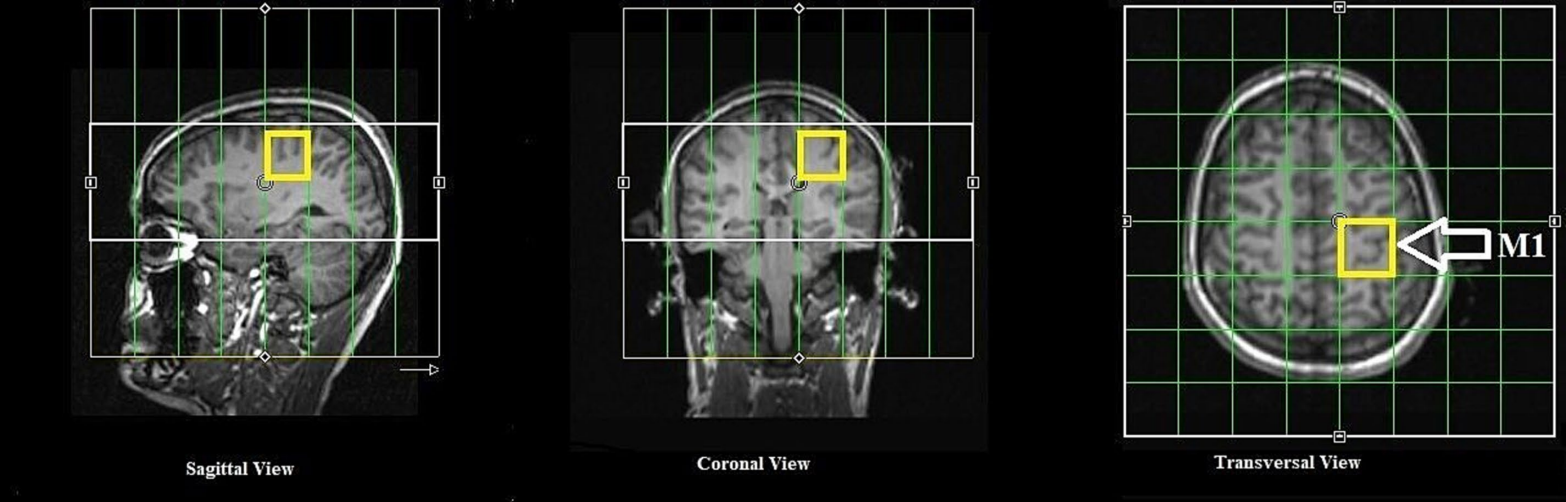
Anatomical MR images in axial, coronal and sagittal slices and the overlaid voxel of interest (in yellow) for ^1^H and ^31^P MRS.

**Figure 3:**
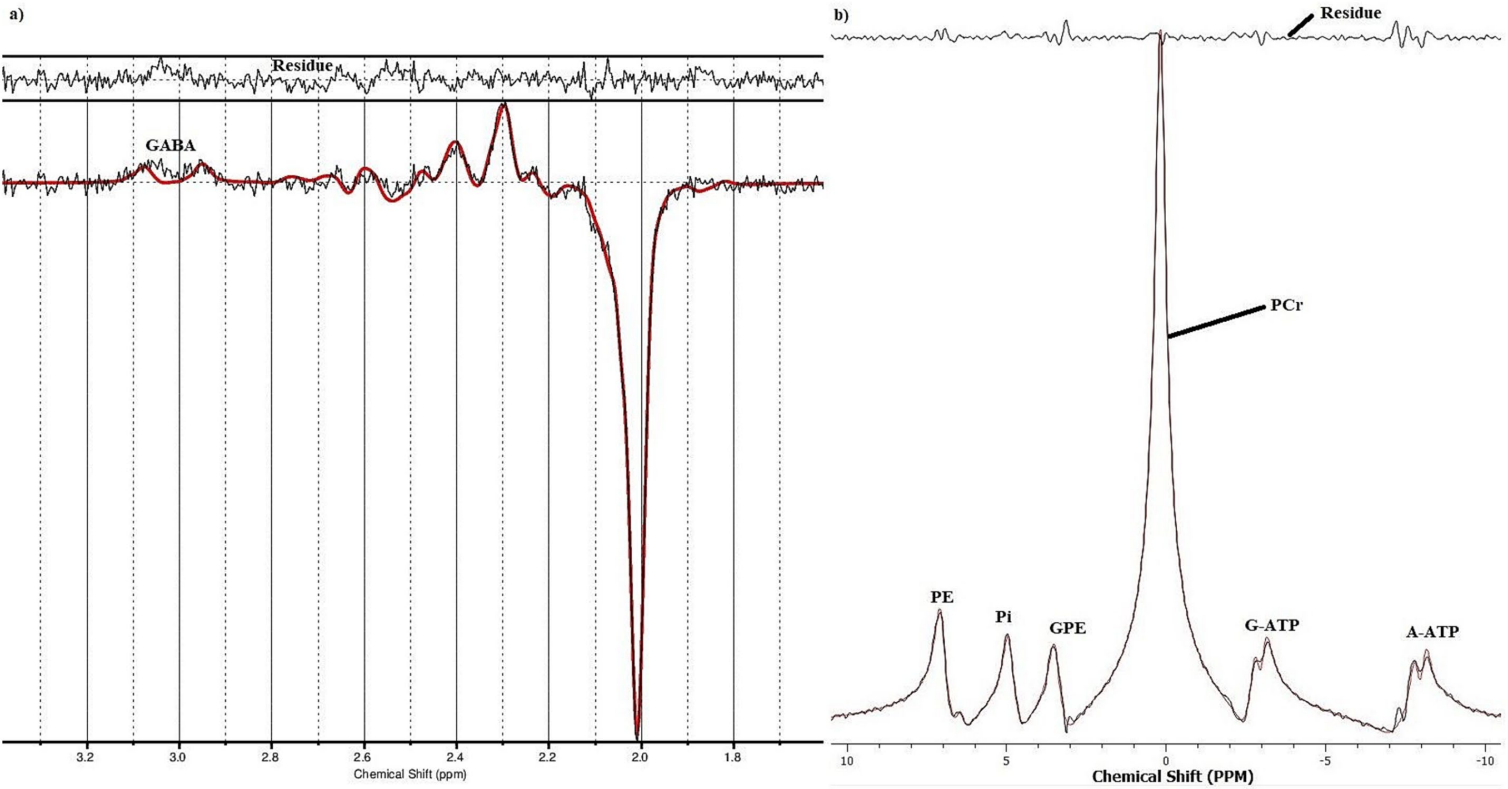
An example of a fitted **(A)** 1H GABA and **(B)** 31P spectrum from a healthy subject.

All MR measurements were carried out on a 3 T PRISMA MRI scanner (Siemens Healthineers, Erlangen, Germany) with a dockable patient bed. A quadrature double-tuned head coil (RAPID Biomedical, Wuerzburg, Germany) was used to achieve both ^1^H and ^31^P acquisitions. Figure 1 shows a tabular representation of the overall experimental design. Each experimental session started with a localizer followed by the acquisition of whole-brain anatomical images using an MP-RAGE MRI sequence [parameters: voxel size = 1.5 × 1.5 × 1.5 mm^3^, repetition time (TR) = 2,000 ms, echo time (TE) = 2.05 ms, inversion time (TI) = 900 ms, acquisition time (TA) = 4:32 min], with RF power and global static magnetic field (B_0_) shimming calibrations. Figure 2 shows the 3D anatomical image used to locate a 30 × 30 × 30 mm^3^ voxel-of-interest (VOI) within the hand-knob area of the left M1. Prior to the MRS measurements, an additional advanced shimming procedure was employed using a FASTEST map MRS sequence (Gruetter and Tkáč, 2000) to improve the B_0_ homogeneity in the selected VOI. A full-width at half-maximum (FWHM) of the ^1^H resonance peak in the VOI was achieved at approximately 15 Hz. A MEGA-PRESS MRS sequence (Mescher et al., 1998; parameters: voxel size = 30 × 30 × 30 mm^3^, TR = 5,350 ms, TE = 68 ms, TA =17:53 min, averages = 96, vector size = 1,024, flip angle = 90^°^) and a 3D chemical shift imaging MRS sequence (Wenger et al., 2020; parameters: voxel size = 30 × 30 × 30 mm^3^, TR = 3,730 ms, TE = 2.3 ms, TA = 14:33 min, averages = 6, vector size = 1,024, flip angle = 90^°^) were applied to acquire ^1^H-GABA and ^31^P spectra, respectively. A standard PRESS MRI sequence with eight averages was also included to record an unedited spectrum for the assessment of the creatine and N-acetylaspartate acid (NAA) linewidths. The nuclear Overhauser effect enhancement technique was employed to improve the quality of the spectra in the ^31^P acquisition. Reference ^1^H and ^31^P spectra as shown in Figure 3 were attained before the application of tDCS (pre-stimulation measurements), which took approximately 35 min. After obtaining the baseline MRS scans, the whole patient table, including the subject and the coil, was undocked from the scanner and moved outside the magnet room for stimulation. The stimulation was delivered for 20 min, during which participants were asked to remain still. Following the stimulation, the patient table was docked back to the MR scanner for the subsequent measurements. Undocking and docking of the table procedure helped to mitigate subject movement between the pre- and post-stimulation MRS measurements. The same MP-RAGE MRI sequence was used post-stimulation to ensure that the location of the VOI was identical to the pre-stimulation position. Two consecutive post-stimulation ^1^H and ^31^P MRS measurements were conducted. The ^1^H MRS measurement was conducted for 17:53 min, and then ^31^P MRS was conducted for 14:33 min twice, in a constant order post-stimulation. All subjects were informed about the duration of the measurement and were asked to remain awake and not to move during the whole experimental procedure.

All ^1^H-GABA-MRS data processing was performed using the LCModel (Provencher, 1993), a spectral quantification tool that fits each spectrum as a weighted linear combination of basis spectra from individual brain metabolites. The difference basis set included GABA, Glu, glutamine, glutathione, and NAA. Based on an 8 × 8 CSI grid, the ^31^P MR spectra from one voxel were analyzed with TARQUIN 4.3.10(Wilson et al., 2011). All data underwent a Fourier transformation, as well as zero and first-order phase correction, and were fitted as linear combinations of the simulated metabolic basis set, including PCr, ATP, Pi, PE, GPC and GPE for ^31^P-MRS. While PCr, Pi, PE, GPC and GPE are observed as singlet Lorentzian peaks, the signals from the α- and γ-ATP were modelled as doublets. However, the signal from ATP-ß was excluded from the analysis due to phasing instabilities (Novak et al., 2014). The quality of the final spectra was assessed using the Cramér-Rao-Lower-Bounds (Rao, 1947) minimum possible variance on a fit parameter. Only data that had Cramér-Rao-Lower-Bounds values of less than ≤20% were included in the analysis (Kreis, 2016; Öz et al., 2014; Peek et al., 2023; Wilson et al., 2019).

### Statistical analysis

In order to investigate the effects of tDCS on the inhibitory neurotransmitter and energy metabolites in the left M1, changes in proton and phosphorus metabolites in pre- and post-stimulation scans were calculated separately to obtain the information about GABA, ATP/Pi and PCr/Pi. Each ratio was normalized (post1-tDCS/pre-tDCS & post2-tDCS/pre-tDCS) with its reference measurement (pre-tDCS). Statistical analysis was conducted with SPSS (version 29.0 Armonk, NY, USA), with *p* = 0.05 set as the significance threshold. A mixed-design Analysis of variance was performed for GABA concentration, ATP/Pi and PCr/Pi metabolites. Stimulation (sham vs. anodal) was considered as the between-subjects factor, and measurement (three measurements: one pre-stimulation and two post-stimulation measurements [post1 and post2]) was considered as the within-subject factor. Paired sample t-test comparisons (*p* > 0.05) were performed in the cases of significant ANOVA results.

## Results

### Effect of tDCS on GABA

**Figure 4:**
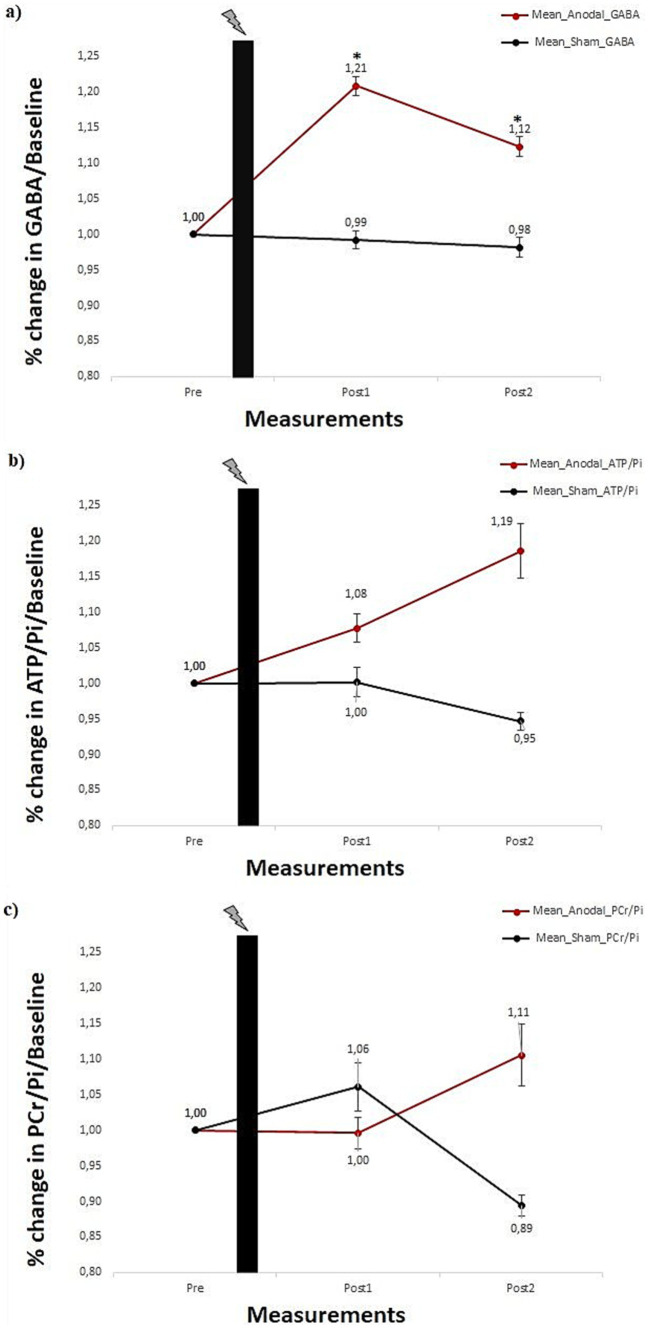
**(A)** Percentage change in mean GABA concentration. **(B)** ATP/Pi ratio, and **(C)** PCr/Pi ratio for anodal and sham stimulation groups, each comprising 22 subjects. Error bars reflect the standard error of the mean. Black plot: anodal/sham stimulation. *Show significances (*p* < 0.05) for a paired sample *t*-test.

For GABA, the main effect of the stimulation was found to be significant [*F*_(1, 42)_ = 4.742, *p* = 0.035]; however, the main effect of the measurement was not significant [*F*_(2, 84)_ = 2.855, *p* = 0.063]. That being said, the stimulation × measurement interaction was significant [*F*_(2,84)_ = 3.387, *p* = 0.038]. Paired sample *t*-test comparisons show that concentration did not change significantly from pre-tDCS measurement to post1- and post2-tDCS measurements [*ts*_(21)_ ≤ 0.277 *ps* > 0.05] for the sham group. Conversely, relative to the pre-tDCS, GABA concentration in the anodal tDCS group significantly increased after 14 min at the post1-tDCS measurement [*ts*_(21)_ = 3.380 *p* = 0.001], and also after 48 min at the post2-tDCS measurement [*ts*_(21)_ = 1.893 *p* = 0.036]. Thus, in the sham group, GABA concentration stayed at the pre-tDCS baseline level across all post-tDCS measurements. In contrast, data from the anodal group suggest an increase in GABA concentration across the two time points in the post-tDCS phase. Figure 4A shows the changes in normalized GABA concentration levels induced by tDCS at pre-tDCS, post1-tDCS and post2-tDCS spectroscopy measurements.

### Effect of tDCS on energy phosphates (ATP/pi & PCr/pi)

For ATP/Pi ratios, neither the main effect of stimulation [*F*_(1,42)_ = 1.573, *p* = 0.217] nor the main effect of the measurement [*F*_(2,84)_ = 0.259, *p* = 0.772] was significant. Furthermore, the interaction stimulation × measurement was also not significant [*F*_(2,84)_ = 0.887, *p* = 0.416]. Figure 4B shows changes in normalized ATP/Pi ratios for pre-tDCS, post1-tDCS and post2-tDCS spectroscopy measurements. For PCr/Pi ratios, neither the main effect of stimulation [*F*_(1,42)_ = 0.218, *p* = 0.643] nor the main effect of the measurement [*F*_(2,84)_ = 0.045, *p* = 0.956] was significant. Additionally, the interaction stimulation × measurement was also not shown to be significant [*F*_(2,84)_ = 852, *p* = 0.430]. Figure 4C shows tDCS-induced effects on normalized PCr/Pi ratios for pre-tDCS, post1-tDCS and post2-tDCS spectroscopy measurements.

## Discussion

The human brain functions as a dynamic system, continually adjusting its metabolic interactions to accommodate the activation status and energy requirements of the entire organism. Therefore, static MRS measurements obtained in single sessions necessarily deliver an incomplete picture of the state of the brain. To address this limitation, our feasibility study demonstrates the acquisition of combined measurements using ^1^H-GABA-MRS, ^31^P-MRS and anodal stimulation to provide unique information relating to adaptive plasticity and energy consumption in the human brain during extended time periods after plasticity-inducing brain stimulation.

Our results indicate a notable increase in GABA levels measured by ^1^H-MRS following 20 min of anodal stimulation over the M1 at both the initial post-tDCS measurement and the second post-tDCS measurement, which contradicts previous reports showing a reduction in GABA levels at these time points (Agboada et al., 2019; Patel et al., 2019). Studies have shown that changes in cortical excitability due to tDCS are calcium-dependent (Grundey et al., 2018; Nitsche et al., 2003) and that calcium concentration within a specific range is required for LTP induction (Lisman, 2001). Therefore, it could be argued that previous studies (Patel et al., 2019; Stagg et al., 2009) utilizing relatively low tDCS intensities and/or short durations might have been operating at the lower limit of the calcium concentrations required for inducing the aforementioned neuroplastic changes. Within the range given in Lisman (2001), increasing calcium concentration should, theoretically, increase the efficacy of LTP induction. Hence, prolonging the duration of stimulation beyond a critical time point may lead to a saturation of the after-effects, possibly caused by calcium overflow (Monte-Silva et al., 2013). Consequently, higher calcium concentration might activate counteracting homeostatic mechanisms, such as the activation of potassium channels or the saturation of NMDA receptors, thereby limiting the amount of plasticity (An et al., 2000; Lau and Zukin, 2007; Misonou et al., 2004). These potential mechanisms require further exploration. To the best of our knowledge, there are very few studies investigating the mechanism underlying these non-linear effects (Batsikadze et al., 2013; Jamil et al., 2017; Monte-Silva et al., 2013). In line with this notion, studies using different non-invasive brain stimulation techniques have also reported non-linear cortical excitability effects post-tDCS (Batsikadze et al., 2013; Kuo et al., 2013) with respect to varying intensity (Moliadze et al., 2012) and duration (Choi et al., 2021; Heimrath et al., 2020; Monte-Silva et al., 2013).

In an apparent contradiction to the results obtained in our study, a study from Agboada et al. (2019) reported that, having applied anodal tDCS with 1 mA current for 20 min, cortical excitation could be subsequently monitored for two hours by measuring motor evoked potentials (MEPs) using TMS. However, the reason for these opposing results might be due to a difference in the study population. Furthermore, the amount of extracellular GABA measured by MRS might not correlate with TMS-induced activity in the GABAergic synapses. As neurotransmitters are active in intracellular and extracellular space (Belelli et al., 2009; Martin and Rimvall, 1993), and MRS is not sensitive enough to differentiate between these sources, it is only possible to speculate about mechanisms that are known to change GABA concentration. Moreover, animal studies conducted by Purpura and McMurtry (1965) have shown that direct stimulation in the deep cortical layers leads to the activation of neurons through cathodal stimulation and deactivation through anodal stimulation. Hence, the longer tDCS protocol used in the present study might have more significant effects on GABAergic neurons, which are less affected under the weaker electric field induced by a shorter stimulation duration. It is also possible that longer tDCS protocols may affect the target neurons as well as neighbouring non-target neurons, which might change the direction of plasticity in the target regions.

The levels of MRS GABA have been linked to behavioural measures in M1, where higher GABA levels were associated with greater inhibition and slower reaction times (Stagg et al., 2011). Changes in GABA levels in the dorsolateral prefrontal cortex (DLPFC) have also been observed in studies on impulsivity, a personality trait associated with various psychiatric disorders in the DSM. Studies have indicated that higher GABA levels in the DLPFC are linked to lower urgency scores, suggesting a role in self-control for the GABA-mediated inhibitory mechanism (Boy et al., 2011). Additionally, research by Kühn et al. (2016) and Yoon et al. (2016) demonstrated that higher GABA levels in the DLPFC and anterior cingulate cortex (Rossini et al., 1994) are associated with less performance degradation under higher working memory load compared to subjects with lower GABA levels. Furthermore, elevated GABA levels in the visual cortex have been associated with improved cognitive and perceptual performance in visual discrimination tasks (Cook et al., 2016; Edden et al., 2009; Porges et al., 2017; Sumner et al., 2010). van Vugt et al. (2020) investigated the influence of GABA modulation on audio perception discrimination and mapping motor responses with perceived sounds. Elevated GABA levels in the left sensorimotor cortex (SM1) during training were associated with enhanced behavioural learning, highlighting the role of GABA modulation in forming unique audio-motor learning tasks. The elevated GABA levels in the ACC during situations involving conflict or uncertainty (Bezalel et al., 2019) contribute to decision-making by maintaining inhibition and preventing excessive excitement. A further study investigated the relationship between GABA levels within the sensorimotor cortex and measures of sensory discrimination and demonstrated that healthy subjects who had higher GABA levels showed greater frequency discrimination (Puts et al., 2011). Higher GABA levels were associated with improved performance in orientation discrimination task as seen in studies of the primary visual cortex (Edden et al., 2009). Similar relationships between a variety of task-specific behaviours and levels of GABAergic inhibition have also been demonstrated in a number of brain regions outside the sensorimotor cortex (Boy et al., 2010; Boy et al., 2011; Jocham et al., 2012; Sumner et al., 2010). This implies that MRS-derived measurements of GABA are behaviorally relevant in a variety of cortical areas, and not limited to M1.GABA modulation is not exclusive to learning and has been observed in memory consolidation as well. For an increase in GABA levels in the occipital lobes following a visual learning task helps in fast memory consolidation and protects against interference (Shibata et al., 2017). The use of Zolpidem, a GABAA agonist, during sleep has been shown to enhance episodic memories, indicating the role of GABA in memory consolidation. The Increased GABA activity during sleep supports memory consolidation (Mednick et al., 2013; Zhang et al., 2020).

Coxon and colleagues discovered that engaging in high-intensity interval exercise resulted in elevated GABA levels in SM1 (Coxon et al., 2018). In a study by Maddock, it was demonstrated that vigorous cycling increased GABA/Cr levels in the primary visual cortex post-exercise (Maddock et al., 2016). This suggests that physical exercise influences regional GABA levels beyond SM1, but the regional specificity of GABA changes due to exercise require further investigation.

It has been well reported that GABA and Glu interact along the same biochemical pathway, as Glu is the primary precursor for GABA synthesis (Petroff, 2002; Rae et al., 2003). Given that GABA is synthesized from the alpha decarboxylation of Glu by glutamic acid decarboxylase (GAD-67), the activity-driven expression of GAD-67 controls GABA synthesis and determines the concentration of GABA in interneurons (Lau and Murthy, 2012). It is possible that anodal tDCS may subtly interfere with GAD-67 activity and consequently affect this pathway, resulting in a change in the GABA concentration. However, we were only able to access the Glx information at 3 T, which combined glutamate and glutamine signals due to their close chemical shift range. A study, for example, using 7 T MRI, might help to investigate long-term glutamate modulation and allow further exploration of the mechanism of excitatory glutamatergic neurons in long-term plasticity.

The observed increase in ATP/Pi and PCr/Pi ratios shown in our study can be partially compared to findings from Binkofski et al. (2011), who reported their first non-significant post-tDCS measurement between the end of tDCS and before around 65^th^ minute of MRS measurement, thus also showing an initial rise in energy followed by a subsequent decline after the 65^th^ minute. The increase in ATP/Pi ratio and PCr/Pi ratio in our study could be attributed to a higher rate of energy phosphate synthesis compared to its consumption as a consequence of the longer tDCS duration. ATP concentration has been observed as a potential modulator of neurotransmission (Miller et al., 1980; Miller et al., 1977), and the neurotransmitter glutamate is a known precursor of GABA synthesis and uses glutamic acid decarboxylase (GAD) to synthesize GABA. At least 50% of GAD is present in the brain as apoenzyme (apoGAD), thereby providing a reservoir of inactive GAD that can be drawn on when additional GABA synthesis is required (Itoh and Uchimura, 1981; Miller et al., 1980; Miller et al., 1977). It seems that GAD plays an important role in regulating the transmitter pool of GABA. Studies indicate that the increase in the ATP/Pi ratio accelerates the formation of apoGAD (Meeley and Martin, 1983; Wu and Martin, 1984), inhibits its activation (Porter and Martin, 1988; Sze et al., 1983) and stabilizes apoGAD against thermal denaturation (Meeley and Martin, 1983; Porter and Martin, 1988). It appears that the activation of GAD is regulated by energy metabolism, and increased neural activity leads to a higher turnover of energy metabolites. Moreover, it is plausible that these energy metabolites play a vital role in linking GAD activity with neuronal activity. Thus, it is plausible that elevated levels of ATP might stimulate the activation of apoGAD, thereby promoting GABA synthesis.

GABA appears to have two major functions in nervous tissue, acting both as a neurotransmitter and as an intermediate in the energy metabolism of GABAergic neurons (Shelp et al., 2012). While much attention is given to the role of GABA as a neurotransmitter, its role as a metabolic intermediate is also important and may help to explain the link between neuronal plasticity and energy metabolism. This metabolic function of GABA arises from its relationship with the tricarboxylic acid (TCA) cycle (Baxter, 1970). The TCA cycle is a series of processes that produce high-energy phosphate, such as ATP, following glycolysis. Three enzymes of GABA pathways, GAD, GABA-a-oxoglutarate transaminase and succinic semialdehyde dehydrogenase, facilitate a bypass known as the GABA shunt. This bypass allows for the circumnavigation of two steps of the TCA cycle, leading to the production of glutamate for GABA synthesis. In this way, GABA is metabolized via the GABA shunt within the TCA cycle, thus emphasizing the role of GABA synthesis in maintaining adequate GABA levels. Based on this neurochemical mechanism, it might be possible that the longer stimulation protocol demands higher energy phosphate synthesis and also higher GABA synthesis, which may work together to regulate the higher demand for energy phosphate and the corresponding GABA synthesis in the brain. Our study demonstrates that anodal tDCS leads to an increase in ATP resynthesis to meet the energy demand, which is similar to the energy regulation observed after physical exercise (Hargreaves and Spriet, 2020). Modulation of energy following physical activity has shown to improve memory, reasoning, planning, motor skill (Nanda et al., 2013; Statton et al., 2015) and promote cognitive functioning in both healthy as well as clinical populations such as stroke, multiple sclerosis and depression (Moriarty et al., 2019; Moriya et al., 2016; Sandroff et al., 2015; Sibley et al., 2006; Vasques et al., 2011; Wang et al., 2016). Physical activity boosts brain-derived neurotrophic factor (BDNF), promoting neuroplasticity (Loprinzi and Frith, 2019), and consequently enhancing memory and learning (Piepmeier and Etnier, 2015), while also offering protection against Alzheimer’s and depression (Bjornebekk et al., 2005; Erickson et al., 2011; Liu and Nusslock, 2018). Elevated BDNF levels are associated with the survival and growth of neurons (McAllister et al., 1999). Lower BDNF levels are linked to ageing and neurodegenerative diseases (Holsinger et al., 2000). These findings reveal the usefulness of physical exercise or alteration of energy regulation as shown in our study can be employed as an auxiliary tool for rehabilitation because it appears to affect the neurological system for learning and skill acquisition.

The results of this study suggest that the new combined approach introduced here has strong implications for the measurement of energy and neural plasticity in both healthy subjects and neuropsychiatric patients (Gobel et al., 2013; Jauch-Chara et al., 2015; Stagg et al., 2011; Stagg and Johansen-Berg, 2013). For instance, psychiatric and metabolic diseases have been found to be interconnected, often acting as a precursor to each other (Estrov et al., 2000; Zuccoli et al., 2017). In this context, combined ^1^H- and ^31^P-MRS together with anodal tDCS may represent a two-pronged approach to better understand impaired glucose metabolism and psychiatric disorders without the need for pharmacological intervention. Furthermore, this approach can be employed in numerous additional experiments aiming to optimize the described experiment from a clinical perspective. For instance, improvements in terms of shortening acquisition time and increasing the signal-to-noise ratio can be achieved at ultra-high field strengths (Pradhan et al., 2015), particularly with the use of double-tuned, multi-channel array coils (Rowland et al., 2020). Additionally, further investigation of the intensity and duration of the stimulation in order to understand the neurochemical mechanisms involved could also extend the impact of the tDCS. Apart from tDCS, future research may explore other transcranial electrical stimulation options, including transcranial alternating current stimulation (tACS), transcranial pulsed current stimulation (tPCS) or transcranial random noise stimulation (tRNS), to investigate neurochemical pathways in the brain. This feasibility study serves as a potential tool for expanding our understanding of the neurochemistry underlying energy metabolism and neuronal plasticity. Moreover, our experimental approach represents an encouraging nonpharmacological alternative to investigate altered neuronal plasticity and energy metabolism in neurological and metabolic disorders.

### Limitations of the study

The current study is limited by the relatively short duration of the post-tDCS measurements, i.e., 67 min following tDCS. This narrow timeframe could be one possible reason for the absence of late significant recovery of energy phosphates. To address this shortcoming and to capture the late energy effects following tDCS, as shown in the study by Binkofski et al. (2011), future studies should aim to measure energy metabolites over a longer time. The follow-up period may not be sufficient to assess the long-term effects of tDCS. Longer-term studies are necessary to determine the durability of the observed changes and their potential implications for clinical applications. Moreover, as this feasibility study is only based on the motor cortex, it cannot be generalized to other brain regions. More studies are needed to explore other brain areas with similar quantitative and statistical investigations.

As studies using identical tDCS protocols have demonstrated different effects in healthy young adults (Alexandersen et al., 2022; Boayue et al., 2020; Willmot et al., 2024), the results obtained from this study are not one-to-one transferable to different age populations or patient groups. However, investigating inter-individual variability was not the aim of the study, and hence, subtle differences between individuals may have been concealed due to the small sample size. For future studies that aim to study inter-individual variability, a larger sample size would be useful for detecting subtle differences. One-to-one comparison with other studies is not possible due to differences in the method, material, acquisition protocols, etc. Further research is needed to investigate the relevance of calcium dynamics at deeper cortical layers and neighbouring brain regions to evaluate the effects of prolonged stimulation protocols. While we have proposed some reasonable explanations and possible molecular mechanisms underlying the aftereffects of prolonged tDCS duration, the precise neurobiological framework regarding neuroplasticity and energy metabolism remains unclear and requires further study.

## Conclusion

We have demonstrated that it is possible to induce long lasting effects by anodal tDCS and measure the corresponding GABA and energy phosphate change in left primary motor cortex using proton and phosphorus magnetic resonance spectroscopy. Hence, this new approach may help to better understand the neurochemical mechanism underlying neurological and metabolic disorders.

## Data Availability

The raw data supporting the conclusions of this article will be made available by the authors, without undue reservation.

## Glossary

ATP: Adenosine triphosphate
B_0_: Static magnetic field
CSI: Chemical shift imaging
FWHM: Fullwidth half maximum
GABA: Gamma amino butyric acid
Glu: Glutamate
Glx: Glutamate-glutamine
GAD: Glutamic acid decarboxylase
LTD: Long-term depression
LTP: Long-term potentiation
M1: Primary motor cortex
MRI: Magnetic resonance imaging
MRS: Magnetic resonance spectroscopy
NAA: N-acetylaspartate acid
PCr: Phosphocreatine
Pi: Inorganic phosphate
GPC: Glycerophosphocholine
GPE: Glycerophosphoethanolamine
TCA: Tricarboxylic acid
tDCS: Transcranial direct current stimulation
TMS: Transcranial magnetic stimulation
MEP: Motor evoked potential
VOI: Volume of interest
TR: Repetition time
TE: Echo time
TA: Acquisition time
PRESS: Point resolved spectroscopy,
MEGAPRESS: Meshcher-garwood point resolved spectroscopy
MDD: Major depressive disorder
DLPFC: Dorsolateral prefrontal cortex
SM1: Sensorimotor cortex
ACC: Anterior cingulate cortex
tACS: Transcranial alternating current stimulation
tPCS: Transcranial pulsed current stimulation
tRNS: Transcranial random noise stimulation
